# Determinants of the uptake of the uncinate process of pancreas in ^68^Ga-DOTATOC PET/CT: a retrospective study

**DOI:** 10.1007/s12020-023-03664-5

**Published:** 2023-12-28

**Authors:** Lucas Jallet, Wadi’ Othmani, Marine Perrier, David Morland

**Affiliations:** 1 Service de Médecine Nucléaire, Institut Godinot, Reims, France; 2 Hépato-Gastroentérologie et Cancérologie digestive, Centre Hospitalier Universitaire de Reims, Reims, France; 3 Laboratoire de Biophysique, UFR de médecine, Université de Reims Champagne-Ardenne, Reims, France; 4 CReSTIC (Centre de Recherche en Sciences et Technologies de l’Information et de la Communication), Université de Reims Champagne-Ardenne, EA 3804 Reims, France

**Keywords:** Somatostatin, PET, Neuroendocrine, Healthy organs

## Abstract

**Purpose:**

an increased uptake of the uncinate process of pancreas (UPP) has been described in about one-third of somatostatin receptor imaging procedures and may hinder image interpretation. The determinants of this uptake are however poorly understood. The aim of this study was to investigate the impact of cold somatostatin analogues (cSA) on UPP ^68^Ga-DOTATOC uptake. Age and diabetic status were also studied.

**Methods:**

all adult patients who performed a ^68^Ga-DOTATOC PET/CT in our center between May 2021 and April 2023 were retrospectively screened. For each one, UPP uptake was visually assessed and measured using SUVmax. Clinical data including cSA medication, age and diabetic status were collected. Univariate and multivariate analyses were conducted using logistic regression. SUVmax comparisons were conducted using a Mann-Whitney Wilcoxon test.

**Results:**

82 patients were included. UPP uptake was significantly lower in patients treated with cSA (OR 0.27, *p* = 0.015 in multivariate analysis), with a lower SUVmax (4.97 vs. 8.81, *p* = 0.001). No significant result was found regarding diabetic status or age.

**Conclusion:**

cold somatostatin analog treatment decreased the physiological UPP uptake in ^68^Ga-DOTATOC PET/CT. This effect could be used to reduce interpretation errors in this location.

## Introduction

Neuroendocrine tumors (NETs) are, in the broader sense, a heterogeneous and rare group of epithelial tumors with neural and endocrine differentiation whose categorization is based more on embryological than anatomical considerations. They gather tumors originating from ectodermal cells (in particular, paragangliomas and pheochromocytomas) and tumors derived from the endodermal cells of the primitive gut [1]. The latter are further subdivided into foregut (mainly pulmonary and pancreatic NETs), midgut (small intestine NETs) and hindgut tumors. NETs share some common features including the expression of somatostatin receptors (SSTR) on their cell membrane, especially when tumors are well-differentiated [2].

SSTR imaging plays a central role in the management of NETs [3]. Initially confined to single photon emitting radiopharmaceuticals with 111In-Pentetreotide, SSTR imaging possibilities have been extended to positron emitters with the arrival of ^68^Ga-labeled peptides, notably ^68^Ga-DOTATATE and ^68^Ga-DOTATOC [4, 5]. In addition to tumor uptake, several physiological uptakes have been described. In particular, an increased uptake of the uncinate process of pancreas (UPP) is described in about 31 to 45% of ^68^Ga-DOTATOC imaging procedures [6, 7] and may represent a pitfall in SSTR imaging, either because it may be confused with a pathological uptake [8] or because it may interfere with the analysis of neighboring structures. The determinants of this uptake are poorly understood. Some authors have suggested a potential influence of diabetes, with contradictory results [9, 10]. Cold somatostatin analogues (cSA), a widely used treatment in small-intestine NETs [11, 12], is known to reduce healthy organs’ ^68^Ga-DOTA peptide uptake without altering tumor visualization [13] but this phenomenon has been studied mainly for the liver and the spleen.

The aim of this study was to assess the effect of cSAs on the UPP uptake in ^68^Ga-DOTATOC in patients with NETs. Age and diabetic status were also studied.

## Methods

### Ethical considerations

This retrospective study was carried out in compliance with the Declaration of Helsinki and the recommendations of the French “Commission Nationale de l’Informatique et des Libertés”. Data collection was declared on the Health Data Hub (N°F20220506115447 - MR004), enabling the computerized management of medical data. Participants were informed of the possibility of using information concerning them, and had the right to object.

### Patients’ selection and data collection

**Fig. 1:**
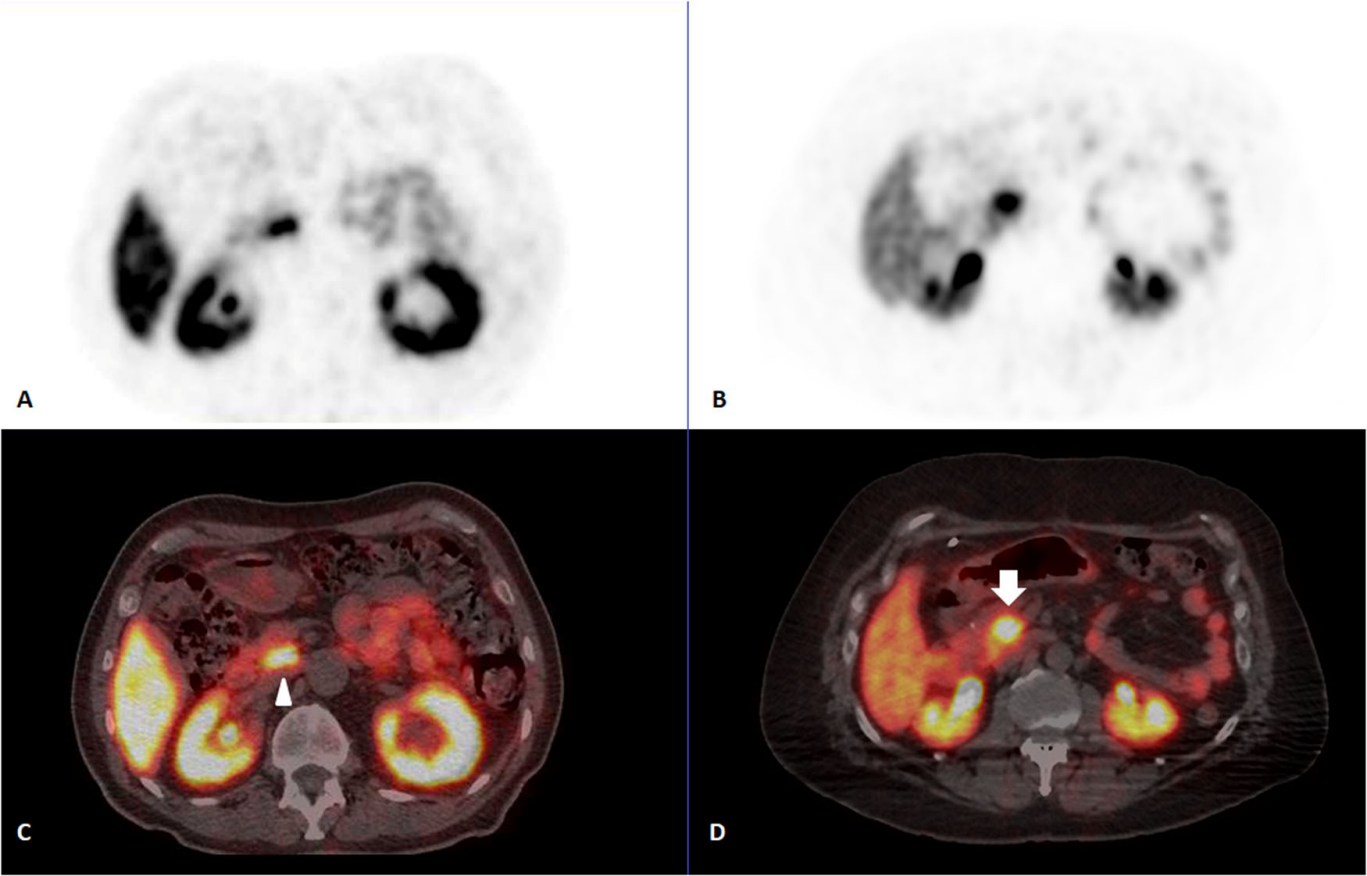
Example of physiological uptake of the uncinate process of the pancreas on 68Ga-DOTATOC PET/CT (arrowhead, PET alone–image **A**, fused PET/CT–image **C**) and tumoral uptake of the uncinate process on 68Ga-DOTATOC PET/CT (plain arrow, PET alone–image **B**, fused PET/CT–image **D**)

All adult patients who performed ^68^Ga-DOTATOC PET/CT in our center between May 2021 and April 2023 were retrospectively screened. Patients for whom the physiological UPP uptake was not measurable were excluded: known UPP primary tumor, increased uptake neighboring lesion preventing UPP assessment, history of pancreaticoduodenectomy. As physiological UPP uptake is sometimes difficult to distinguish from malignant lesions [14], follow-up examinations and/or histopathological results were used to deal with the issue. An example is displayed (Fig. 1). Clinical and imaging data were collected for each patient: age, sex, diabetes mellitus, ongoing cSA medication, histology of the primary tumor and grade according to the 2019 WHO classification [15] UPP increased uptake visualization (yes/no), uptake measurements (UPP, liver, blood pool, tumor). Additionally, the type, periodicity and date of last administration of cSA was retrieved.

### SSTR imaging and uptake measurements

A Discovery 710 PET/CT system was used (General Electrics, Milwaukee, USA). Images, ranging from head to mid-thigh, were acquired 60 min after an intravenous administration of 3 MBq/kg of ^68^Ga-DOTATOC, using an acquisition time of 120 seconds per step. Images were reconstructed with an iterative ordered subset expectation maximization algorithm (OSEM: 24 subsets, 2 iterations; Butterworth post-filtering with a 6.4 mm cut-off) on a 256 × 256 matrix (voxel size: 2.73 × 2.73 × 3.27 mm^3^). PET/CT scans were displayed and analyzed on a dedicated interpretation console (AW server, General Electrics, Milwaukee, USA). SUVmax was preferred to SUVmean for the reason of reproducibility. UPP maximal standard uptake value (SUVmax) was measured using a manually positioned cubic enclosing region of interest (ROI). Liver SUVmax and blood pool SUVmax were measured using the same type of ROI positioned respectively in the right liver and the descending aorta, away from metastatic lesions, if any. Tumor SUVmax was defined as the highest tumoral uptake whether it corresponded to the primary tumor or a metastasis in a given patient. SUVmax measurements were performed by two of the authors (LJ and DM), and any disagreement was solved by consensus.

### Statistical analysis

Patient’s characteristics were described using number and percentage for categorical variables and mean and standard deviation for quantitative variables. Comparisons between patients with or without cSAs were performed using Fisher exact test or Mann-Whitney Wilcoxon test when appropriate.

Univariate analyses based on logistic regression were conducted between UPP visualization (binary variable) and age, cSA and diabetic status. Only factors with a *p*-value of less than 0.20 were included in the multivariate analysis. Odd ratios (OR) are presented with their 95% confidence intervals (95%CI). Pearson correlation coefficient between UPP SUVmax and delay between PET and last cSA administration was performed. A *p* value < 0.05 was considered as significant. Analyses were performed using XLSTAT (Addinsoft).

## Results

### Included patients’ characteristics

**Fig. 2:**
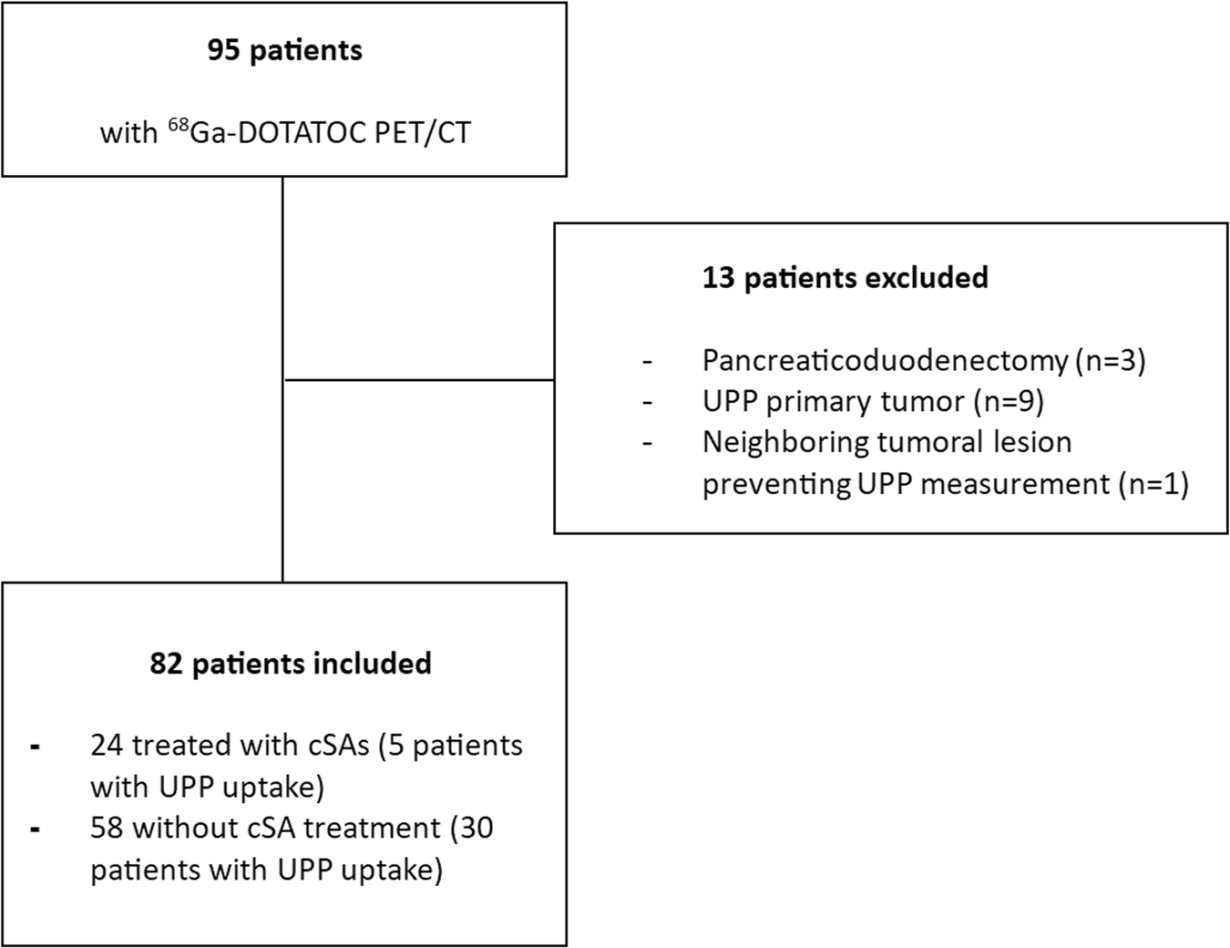
Patients’ flowchart. UPP uncinate process of pancreas, cSA cold somatostatin analogs

A total of 95 patients underwent a ^68^Ga-DOTATOC PET/CT in our center (Fig. 2). Thirteen (13,7%) were excluded: 3 patients had a history of pancreaticoduodenectomy, 9 patients had a pathological tumoral uptake of the UPP and one had a neighboring tumoral lesion preventing UPP measurement. A total of 82 patients were finally included. Among them, 24 (29.3%) patients were treated with cSAs and 26 (31.7%) had a history of diabetes mellitus. Baseline patients’ characteristics are available in Table 1.

**Table 1:** Baseline characteristics of included patients

Characteristics, *n* (%)	Total *N* = 82	Treated with cSAs *N* = 24	Without cSAs *N* = 58	*p* value
**Sex**				
Male	39 (47.6%)	9 (37.5%)	30 (51.7%)	0.33
Female	43 (52.4%)	15 (62.5%)	28 (48.3%)	
**Age, years** (mean, SD)	61.4 (15.5)	69.3 (11.8)	58.1 (15.8)	0.004*
**Primary tumor**				
Pancreas	28 (34.1%)	2 (8.3%)	26 (44.8%)	<0.001*
Small Intestine	23 (28.0%)	19 (79.2%)	4 (6.9%)	
Lung	5 (6.1%)	1 (4.2%)	4 (6.9%)	
Paraganglioma	7 (8.5%)	0 (0.0%)	7 (12.1%)	
Other	19 (23.2%)	2 (8.3%)	17 (29.3%)	
**Grade**				
I	27 (32.9%)	10 (41.7%)	17 (29.3%)	0.028*
II	25 (30.5%)	11 (45.8%)	14 (24.1%)	
III	4 (4.9%)	0 (0.0%)	4 (6.9%)	
NA	26 (31.7%)	3 (12.5%)	23 (39.7%)	
**Diabetes**	26 (31.7%)	4 (16.7%)	22 (37.9%)	0.072

*NA* not available, as grading is only estimated in endodermal NETs (pancreas, small intestine and lung).*p < 0.05

Patients treated with cSAs tended to be older (69.3 vs. 58.1 years, *p* = 0.004) with an over-representation of small intestine primary NETs (79.2%) and grade I–II (87.5%). No statistically significant differences were noted regarding sex ratio.

### Determinants of the visualization of an increased uptake in the UPP

In univariate analysis, an ongoing cSA treatment was significantly associated with lower UPP uptake (OR 0.25, *p* = 0.013). In the subgroup treated with cSAs, 5/24 (20.8%) patients had an UPP-increased uptake versus 30/58 (51.7%) in the subgroup without cSAs. Neither age nor diabetic status was associated (*p* = 0.955 and *p* = 0.165 respectively).

In multivariate analysis, cSA treatment remained significant (OR 0.27, *p* = 0.015). Results are presented in Table 2.

**Table 2:** Univariate and multivariate analyses: determinants of uncinate process visualization

	Univariate analysis		Multivariate analysis	
	OR (95% CI)	*p*-value (Wald)	OR (95% CI)	*p*-value (Wald)
Age	1.001 [0.97–1.03]	0.955	–	–
Diabetes	1.944 [0.76–5.00]	0.165	1.567 [0.59–4.18]	0.370
cSA	0.246 [0.08–0.75]	0.013*	0.268 [0.09–0.83]	0.015*

*cSA* cold somatostatin analogs treatment. *p < 0.05

### Quantitative measurements

The complete results are presented in Table 3. Patients treated with cSAs had a significantly lower UPP uptake (4.97 vs. 8.81, *p* = 0.001). Hepatic uptake was also significantly lower during cSAs treatment (6.43 vs. 10.12, *p* < 0.001). Tumor uptake was slightly lower under cSAs, but the difference was not statistically significant. Blood uptake was significantly higher under cSA treatment (1.76 vs. 1.40 *p* = 0.016).

**Table 3:** SUVmax of healthy organs and tumor lesions during cSA treatment

	Treated with cSA *N* = 24	Without cSA *N* = 58	Ratio With/Without	*p*-value
**SUVmax mean (SD)**				
UPP	4.97 (2.74)	8.81 (5.04)	0.56	0.001*
Liver	6.43 (2.26)	10.12 (2.95)	0.64	<0.001*
Blood	1.76 (0.79)	1.40 (0.94)	1.26	0.016*
Tumor	27.52 (11.88)	38.26 (29.68)	0.72	0.224

*UPP* uncinate process of pancreas.*p < 0.05

### cSA administration and correlation with UPP SUVmax

Among the 24 patients treated with long-acting release cSAs, 18 (75%) were treated with Lanreotide and 6 (25%) with octreotide; cSAs were administered every 28 days for 10 patients and every 14 days for 10 patients. The mean delay between the last administration and the PET was 12 days +/– 9. No significant correlation was found between the delay and the UPP SUVmax (*p* = 0.09). The results are presented in Table 4.

**Table 4:** Cold somatostatin analogue treatments and correlation with uncinate process SUVmax

**Long-acting release analogue:** ***n*** **(%)**	
Octreotide (30 mg)	6 (25%)
Lanreotide (120 mg)	17 (71%)
Lanreotide (60 mg)	1 (4%)
Not available	0 (0%)
**Periodicity:** ***n*** **(%)**	
Every 28 days	10 (42%)
Every 14 days	10 (42%)
Not available	4 (16%)
**Delay PET – last administration of analogues**	
Mean (standard deviation) in days	12 (9)
Not available	2
* Correlation with uncinate process SUVmax*	
Coefficient [95% confidence interval]	−0.39 [−0.71;0.06]
*p*-value	*p* = 0.09

## Discussion

An increased uptake of the UPP has been described in about one-third of ^68^Ga-DOTA-peptides PET/CT [6, 7, 16], similar to that reported with older tracers such as ^111^In-DTPA-octreotide [9]. This uptake has been reported to be due to pancreatic polypeptide cell hyperplasia [8, 9], a particular cell subtype known to be restricted to the uncinate process in the pancreas [17].

Kroiss et al. [14] suggested at first that UPP SUVmax could help differentiate the benign or malignant origin of this uptake with an SUVmax of 33.6 ± 14.3 for NETs and 10.5 ± 4.1 for physiological uptake. However, the physiological uptake group included patients without increased uptake of the UPP considering the reported SUVmax range (2.9 to 28.7). The difference between increased physiological UPP uptake and pathological uptake is therefore more subtle. This physiological uptake may hinder SSTR imaging interpretation.

Our study showed that the administration of cSAs was associated with decreased physiological UPP uptake in ^68^Ga-DOTATOC PET/CT. The mean SUVmax value was significantly lower in the UPP when patients received cSA. A recent systematic review has shown that cSAs, acting on the same target as ^68^Ga-DOTA-peptides, led to a decrease in healthy organ uptake [13]. Among the included studies, only the study by Jahn et al. [18] reported changes in pancreatic uptake, with a decrease of SUVmax of around 50% under cSAs. We confirmed this result with a similar decrease factor. No significant correlation was found between the delay PET--last-administration of cSA and SUVmax. As previously described by Morland et al. [13], cSAs also led to a significant decrease of hepatic SUVmax in our study. Conversely, the mean blood pool SUVmax value was significantly higher: the residual tracer concentration in the blood is probably increased by the lack of tracer uptake by healthy organs.

The hypothesis of a link between UPP uptake and the presence of diabetes mellitus quickly arose in the literature: while a negative correlation was reported by Oh et al. [19] between blood glucose levels and UPP uptake, the diabetic status seems to have no impact [10]. Similarly, we found no impact of diabetes either. Age also seemed to have no influence, as previously reported [10].

Nowadays, cSAs are particularly used in well-differentiated small intestinal (or midgut) NETs and little or not at all in other indications, explaining the over-representation of this type of tumor in the cSA-treated subgroup. Long-administration of cSAs inhibits indeed tumor growth and prolongs progression-free survival in patients with well-differentiated gastro-entero-pancreatic NETs [11, 12, 20]. However, in light of our results, the question of cSAs administration in other indications to reduce physiological uptake and improve imaging quality may arise. A single intravenous administration of short-acting cSAs 15 min prior to 68-DOTA-peptide administration may be sufficient to produce a decrease in physiological uptake [18]. The risk of altering tumor uptake with such a protocol seems to have been ruled out [13]. Although tumor uptake seems to decrease slightly during cSAs treatment, the tumor/healthy organ contrast remains increased overall [13]. The only downside might be the proximity of large vessels given the increase in blood uptake. Prospective multicentric studies will be needed.

Some limitations should be acknowledged. First, the low number of patients especially in the subgroup of patients receiving cSA treatment, could lead to a lack of power, particularly regarding the role of diabetes mellitus. We did not consider blood glucose levels, as they were not measured in routine for this type of examination.

## Conclusion

Cold somatostatin analog treatment decreased the physiological uptake of the uncinate process of pancreas in ^68^Ga-DOTATOC. The question of use of an unlabeled pre-dose of short-acting somatostatin analog when the PET/CT examination is requested for a pancreatic lesion is worth asking and will require further studies.
